# Infant crying problems related to maternal depressive and anxiety symptoms during pregnancy: a prospective cohort study

**DOI:** 10.1186/s12884-021-04252-z

**Published:** 2021-11-17

**Authors:** Tabitha Krogh Ölmestig, Volkert Siersma, Anna Rubach Birkmose, Jakob Kragstrup, Ruth Kirk Ertmann

**Affiliations:** The Research Unit for General Practice and Section of General Practice, Department of Public Health, University of Copenhagen, Øster Farimagsgade 5, 1353 Copenhagen K, Denmark

**Keywords:** Infant crying, Crying problems, Depressive symptoms, Anxiety symptoms, Pregnancy

## Abstract

**Background:**

Infant crying may cause concerns among new parents and is a frequent reason for seeking help from their general practitioner (GP). The etiology of crying problems in infancy is not fully understood, but recent studies have found associations with maternal mental factors. It is well-established that postpartum depression is related to infant crying problems while the influence of maternal mental problems in pregnancy on infant crying is less investigated. We aimed to explore whether maternal depressive symptoms or maternal anxiety during pregnancy were related to crying problems by the newborn child.

**Methods:**

In this prospective cohort study, 1290 pregnant women and their newborn children were followed throughout pregnancy until 8 weeks postpartum. Depressive symptoms and anxiety symptoms were assessed three times during pregnancy and again 8 weeks postpartum with the Major Depressive Inventory (MDI) and the Anxiety Symptoms Scale (ASS). Eight weeks postpartum the mothers were also asked whether their child cried in a way they found problematic. Multivariable regression was used to assess the association between depressive and anxiety symptoms during pregnancy and crying problems, and to adjust for potential confounders.

**Results:**

We found statistically significant associations between high scores of depressive symptoms and anxiety symptoms in pregnancy and infant crying problems. Previously reported strong associations postpartum between depressive symptoms, anxiety symptoms and infant crying problems were also observed in the present data.

**Conclusion:**

These results indicate that mental problems during pregnancy are associated with having a child with crying problems after birth. If more focus is given to maternal mental problems during pregnancy, the healthcare system might be able to detect and help these women, which would be beneficial for both mother and child.

## Background

Crying is a normal and necessary way for the infant to communicate basic needs. However, there are big differences between infants in the amount of crying and, consequently, the concerns of the parents about the crying [1]. Concerns about crying and the stressful and helpless situation experienced by the parents are frequent reasons for seeking advice and help from health professionals [2–4] and trying alternative treatments such as chiropractic therapy [5]. Excessive and prolonged infant crying has even been associated with child abuse and shaken baby syndrome [6–8].

It has been hypothesised that excessive infant crying, even though remitting after a few months, can cause long term behavioral, emotional and psychosocial effects in childhood, such as externalizing problems, internalizing problems, attention-deficit/hyperactivity disorder (ADHD), conduct disorder, and mood problems [9–12].

In the literature and in clinical practice there is no consensus on the definition of problematic infant crying, and the terms vary. Abnormal crying may be referred to as “excessive crying”, “persistent crying”, or “infantile colic”. One suggested definition of the abnormal is crying for more than 3 h per day, on at least 3 days in a week (the modified Wessel criteria) [13]. Other definitions include the need for seeking professional help [14] or the parents’ perception of the crying being “a lot” or the crying being experienced as a problem [15–17].

There are many possible causes of infant crying problems, none definite, however [15, 18]. Focus has been given to conditions in the child causing excessive crying, such as intestinal immaturity, cow’s milk allergy [19], and intestinal microbiota composition [20]. A number of studies have raised the question, whether the cause could be found in maternal factors. Infant crying problems have been associated with low maternal age and educational level [21]. Some researchers agree that crying problems derive from the complex mother-infant dyad [22, 23], and it is well-established that maternal postpartum depression and infant crying influence each other. This has led to investigations on the association between maternal mental problems during pregnancy and infant crying.

Systematic reviews on this topic have in general found associations between maternal mental problems (stress, anxiety or depression) in pregnancy and infant regulatory problems including crying [24–26]. There is, thought, some conflicting evidence whether the association is strongest for depressive symptoms or anxiety symptoms, where some studies did not find any association of excessive crying with maternal depression [27, 28] or anxiety [29]. Few studies have investigated this association between maternal mental problems during pregnancy and excessive infant crying prospectively [28–35]. However, there is still a lack of prospective studies investigating this association, and the relative importance of maternal depressive and anxiety symptoms is not well described [36].

Therefore, we aimed to explore whether maternal depressive symptoms or maternal anxiety symptoms during pregnancy were related to crying problems by the newborn child. We hypothesized that women with high depressive scores and high anxiety scores would be in greater risk of having a child with crying problems. A priori, we also expected that this risk would be more pronounced for women with symptoms in late pregnancy.

## Material and methods

### Design

This study is part of a Danish prospective cohort study, where 1290 pregnant women, from region Zealand and the Capital region, participated by completing questionnaires throughout their pregnancies until a postpartum consultation 8 weeks after birth. The women gave the research group informed consent to insight into their pregnancy health records [37] and completed questionnaires.

### Setting

The healthcare system in Denmark is tax funded, and care, in both the primary and secondary healthcare system, is free of charge for the patient. The vast majority of the population in Denmark (99%) is registered with a general practitioner (GP), who functions as a gatekeeper to secondary health care. The GPs provide at least three consultations during pregnancy and one consultation postpartum, which most pregnant women attend. The first consultation is offered in gestational weeks 6–10 to all women who wish to go ahead with their pregnancy. A structured pregnancy health record is established and sent to midwives and hospital departments. The second and third prenatal consultations usually take place around weeks 25 and 32 of gestation and the postpartum examination 8 weeks after birth.

### Population

**Fig. 1:**
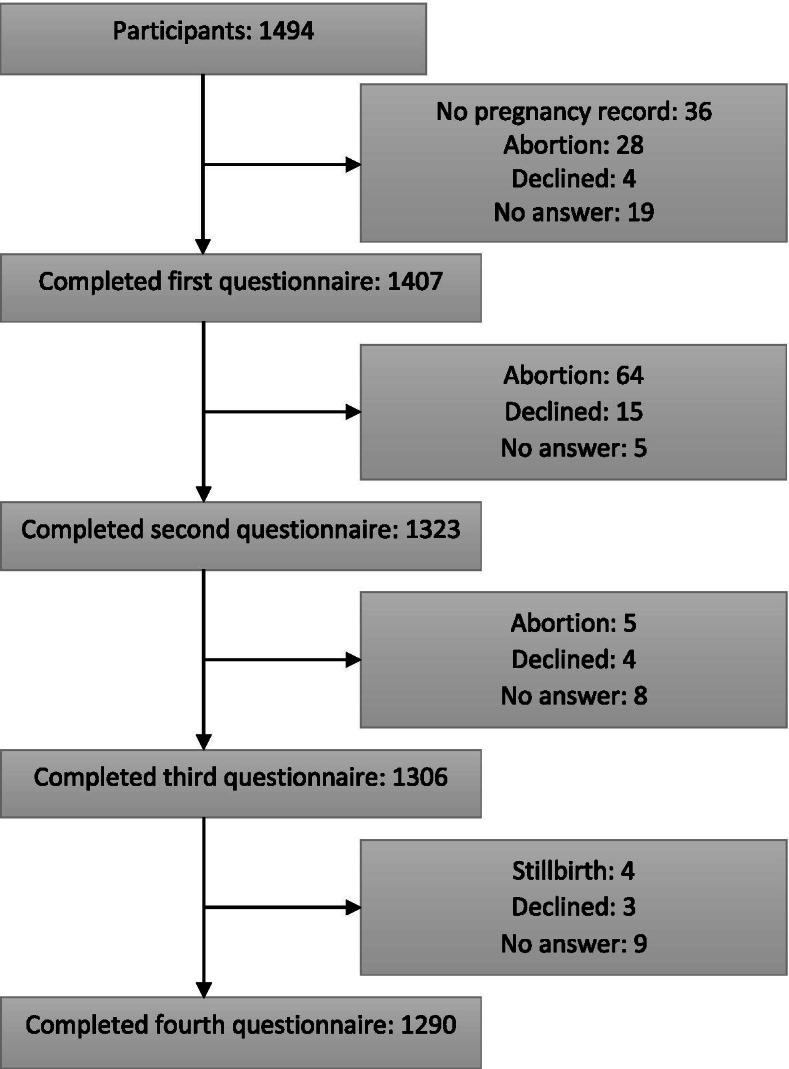
Flowchart of the inclusion process. This figure illustrates the number of participants, who were included in the study, and the reasons for exclusion

The pregnant women were recruited through their GP. The participating GPs came from two of the five Danish regions (The Capital Region and Region Zealand). They were organised into 53 geographical units, from which 19 units were randomly selected (306 practices). Practices were individually contacted and invited to participate in the study, and 192 practices agreed to participate, comprising a total of 294 GPs in single-handed or partnership practices. Eligible for inclusion in the project were all pregnant women booking an appointment for the first prenatal care consultation at one of the participating practices in the inclusion period (1 April 2015 to 15 August 2016). They were included after signing a consent form. Women were excluded, if they did not complete the electronic questionnaires (all in Danish) (41 women), if they withdrew consent (26 women), if the pregnancy ended in abortion (97 women), if the child was stillborn (4 women), or if the Pregnancy Health Record was lost (36 women). Informed consent to participate was obtained from 1494 pregnant women and 1290 completed the study. The majority of dropouts was due to abortion (Fig. 1).

### Data collection

Data for the present article were collected from the women’s Pregnancy Health Record (a standard national two-page questionnaire) and from electronic patient questionnaires (sent and answered on the web by means of SurveyXact). The Pregnancy Health Record was completed by the GP at the first prenatal consultation and sent to midwives and the expected place of birth. The electronic patient questionnaires, which were designed for this study, were sent to the women in first, second, and third trimester and again 8 weeks postpartum after the regular consultations with the GP.

### Measures

#### Infant crying problems

Maternal perception of the infant’s crying was assessed by a questionnaire 8 weeks postpartum, where the mothers were asked about their infant’s crying during the past week: Is the crying considered a problem? (Response categories: “Yes, a major problem”, “Yes, a minor problem”, “No problem”). Infant crying problems were defined as present, if the mother answered that the crying was considered a minor or major problem.

#### Depressive symptoms during pregnancy and postpartum

The Major Depression Inventory (MDI) was part of the questionnaires completed by the women in the first, second, and third trimester, and 8 weeks postpartum. The MDI covers the ICD-10 (WHO 1993) and the DSM-5 (APA 2013) symptoms of clinical depression [38]. The MDI contains 10 items measured on a 6-point scale from 0 (at no time) to 5 (all the time) with a time frame of the past 2 weeks. The total score of the MDI has a theoretical range from 0 (no depression) to 50 (extreme depression). In this study we used the MDI classification based on ICD-10: a total score below 21 is not indicative of depressive symptoms, a total score of 21–25 indicates mild depressive symptoms, a total score of 26–30 indicates moderate depressive symptoms, and a total score of 31 or above indicates severe depressive symptoms [38]. MDI has been demonstrated to have good general validity and reliability [39, 40].

#### Anxiety symptoms during pregnancy and postpartum

Concurrently, the participants completed the Anxiety Symptom Scale (ASS) [41] in the first, second, and third trimester and 8 weeks postpartum. The ASS is used as a screening tool, analogue to MDI, in clinical settings. The ASS comprises 10 items measuring various types of anxiety and their severity, each scoring 0 to 5. Hence, ASS has a theoretical range from 0 (no anxiety) to 50 (extreme anxiety), and a score of 3 is defined as clinical threshold. In this study we used the classification where a total score of 0–2 indicates no anxiety symptoms, a total score of 3–5 indicates mild anxiety symptoms, a total score of 6–9 indicates moderate anxiety symptoms, and a total score of 10 or above indicates severe anxiety symptoms [41]. The ASS is a shorter version of the well validated 20-item State-Trait Anxiety Inventory (STAI). It was developed by Per Bech [40] primarily for clinical use. It has good face validity, but psychometric properties of e.g. subscales have not been documented, and only the total score is, therefore, used in the present study as a general indicator of anxiety symptoms.

The women’s age was divided into the groups: Age (< 25, 26–30, 31–35, > 36 years old). From the pregnancy health records, the following parameters were recorded: parity (nullipara (never delivered a child or still-born prior to present pregnancy), primipara (one previous delivery), multipara (more than one previous delivery)), and previous psychiatric disorders (psychological difficulties requiring treatment, psychiatric diagnosis). Other general information about the women was obtained from the questionnaires. It contained questions concerning socioeconomic factors, lifestyle habits, and mental health. Socioeconomic factors: marital status (married, cohabiting, single), children living at home at start of pregnancy (yes, no), years of completed or ongoing higher education defined as above high school level (no higher education, 3 years, 3–4 years, > 5 years), and income of household (< 39,999 EUR, 40,000–80,299 EUR, 80,300–120,455 EUR, > 120,456 EUR, decline to answer). The women were asked about their occupation, defined as their main source of income (employed, student, unemployed, sick leave, other). In Denmark, students may receive state funded educational support, and unemployed get various subsidies. The women also provided information about lifestyle habits: smoking during pregnancy (yes, no), drinking alcohol during pregnancy (yes, no) and drug use during pregnancy (yes, no). Furthermore, they were asked about previous psychological difficulties (no, yes but did not seek treatment, yes and received treatment).

### Statistical analyses

Characteristics of the study population were compared between mothers of babies with crying problems and mothers of babies without crying problems using chi-squared tests. Logistic regression analyses were used to test the association between depressive symptoms during pregnancy and crying problems 8 weeks postpartum and the association between anxiety symptoms during pregnancy and crying problems. These associations were assessed as odds ratios (ORs), first unadjusted and then adjusted for age, parity, socioeconomic factors, chronic diseases, and psychological problems prior to pregnancy, as these could possibly have confounding effects. The level of significance was set to 5%. The statistical analyses were performed with SAS version 9.4 (SAS Institute, Cary, NC, USA).

## Results

Informed consent to participate was obtained from 1494 pregnant women and 1290 (86,3%) completed the study. The majority of dropouts was due to abortion (Fig. 1).

At 8 weeks postpartum, the prevalence of infant crying problems were 18.2% (235 infants). Table 1 compares socioeconomic characteristics and lifestyle habits between mothers of infants with crying problems and mothers of infants without. Crying problems were seen less often if the mother had other children, while the other examined factors showed no statistically significant relationships.

**Table 1:** Characteristics of study population

	Mothers of infants with crying problems		Mothers of infants without crying problems				
	N	(%)	N	(%)	df	X^**2**^-test value	***p***-value
Total	235	(18.2)	1055	(81.8)			
Age					3	3.669	0.300
< 25 years old	45	(19.2)	184	(17.5)			
26–30 years old	90	(38.3)	392	(37.3)			
31–35 years old	75	(31.9)	312	(29.7)			
> 36 years old	25	(10.6)	162	(15.4)			
Civil status					2	0.884	0.643
Living with partner	223	(94.9)	1002	(95.0)			
Living with others	4	(1.7)	11	(1.0)			
Living alone	8	(3.4)	42	(4.0)			
Other children living at home					1	9.717	0.002
No	118	(50.2)	413	(39.2)			
Yes	117	(49.8)	642	(60.9)			
Parity					2	10.630	0.005
Nullipara	127	(54.0)	453	(42.9)			
Primipara	80	(34.0)	414	(39.2)			
Multipara	28	(11.9)	188	(17.8)			
Educational level					3	2.730	0.435
No higher education	50	(21.3)	277	(26.3)			
Education - 3 years	32	(13.6)	135	(12.8)			
Education - 4 or 5 years	97	(41.3)	395	(37.4)			
Education > 5 years	56	(23.8)	248	(23.5)			
Occupation					4	0.797	0.939
Employed	173	(73.6)	802	(76.0)			
Student	34	(14.5)	142	(13.5)			
Unemployed	12	(5.1)	52	(4.9)			
Sick leave	5	(2.1)	19	(1.8)			
Other	11	(4.7)	40	(3.8)			
Current household income					5	3.537	0.472
< 299,000 DKK	33	(14.0)	120	(11.4)			
300,000–599,000 DKK	64	(27.2)	325	(30.8)			
600,000–899,000 DKK	80	(34.0)	355	(33.7)			
> 900,000 DKK	21	(8.9)	115	(10.9)			
Decline to answer	37	(15.7)	140	(13.3)			
Smoking during pregnancy					1	2.463	0.117
No	222	(95.7)	975	(92.9)			
Yes	10	(4.3)	75	(7.1)			
Drinking during pregnancy					1	0.101	0.751
No	231	(99.1)	1043	(99.3)			
Yes	2	(0.9)	7	(0.7)			
Drugs during pregnancy					1	0.663	0.416
No	228	(100)	1031	(99.7)			
Yes	0	(0.0)	3	(0.3)			

This table shows socioeconomic characteristics and lifestyle habits of mothers of infants with and without crying problems

Table 2 shows the analysis of the relationship between the mother’s depressive symptoms (expressed as MDI score) during and after pregnancy and crying problems in the child. An association was observed between a high depressive score (MDI >  31) in first trimester of pregnancy and crying problems, but it became statistically insignificant after adjustments for previous psychological problems. Mothers with severe depressive symptoms (MDI >  31) in the last trimester of pregnancy were more likely to get a baby with crying problems. A similar association was found for high scores postpartum. These associations remained statistically significant even after adjustment for socioeconomic factors, chronic diseases and previous psychological problems of the mothers. No clear relationship was found for midrange MDI scores in late pregnancy or postpartum.

**Table 2:** Relationship between infant crying problems and maternal depressive symptoms

	Mothers of infants with crying problems		Mothers of infants without crying problems		Chi-sq test	Unadjusted		Adjusted for socioeconomics		Adjusted for chronic diseases		Adjusted for previous psychological difficulties		Adjusted for all blocks	
	***N*** = 235		***N*** = 1055												
MDI score	N	(%)	N	(%)	***P***-value	OR	[95%CI]	OR	[95%CI]	OR	[95%CI]	OR	[95%CI]	OR	[95%CI]
**First trimester**					0.065										
0–20	188	(80.0)	905	(85.8)		ref		ref		ref		ref		ref	
21–25	23	(9.8)	68	(6.5)		1.628	[0.989; 2.680]	1.694	[1.002; 2.865]	1.652	[0.987; 2.763]	1.505	[0.908; 2.495]	1.580	[0.913; 2.734]
26–30	11	(4.7)	50	(4.7)		1.059	[0.541; 2.072]	1.119	[0.546; 2.294]	0.991	[0.487; 2.020]	0.965	[0.490; 1.902]	0.941	[0.437; 2.028]
> 31	13	(5.5)	32	(5.5)		1.956	[1.007; 3.797]	1.981	[0.995; 3.947]	1.970	[1.008; 3.853]	1.718	[0.875; 3.374]	1.687	[0.829; 3.433]
**Second trimester**					0.330										
0–20	195	(83.0)	917	(86.9)		ref		ref		ref		ref		ref	
21–25	15	(6.4)	61	(5.8)		1.156	[0.644; 2.077]	1.288	[0.698; 2.377]	1.152	[0.627; 2.116]	1.105	[0.613; 1.991]	1.203	[0.637; 2.270]
26–30	13	(5.5)	44	(4.2)		1.389	[0.734; 2.629]	1.450	[0.750; 2.804]	1.412	[0.739; 2.699]	1.269	[0.666; 2.420]	1.366	[0.693; 2.691]
> 31	12	(5.1)	33	(3.1)		1.710	[0.868; 3.371]	1.850	[0.911; 3.758]	1.682	[0.826; 3.428]	1.490	[0.747; 2.975]	1.539	[0.729; 3.248]
**Third trimester**					0.002										
0–20	205	(87.2)	965	(91.5)		ref		ref		ref		ref		ref	
21–25	13	(5.5)	52	(4.9)		1.177	[0.629; 2.201]	1.198	[0.628; 2.284]	1.248	[0.662, 2.351]	1.064	[0.564; 2.006]	1.132	[0.585; 2.192]
26–30	6	(2.6)	27	(2.6)		1.046	[0.426; 2.566]	1.005	[0.400; 2.528]	1.124	[0.452; 2.793]	0.871	[0.347; 2.181]	0.920	[0.354; 2.392]
> 31	11	(4.7)	11	(4.7)		4.707	[2.014;11.005]	5.805	[2.405;14.010]	4.938	[2.013;12.115]	4.155	[1.760; 9.808]	5.476	[2.157; 13.901]
**Postpartum**					0.001										
0–20	210	(89.4)	1008	(95.6)		ref		ref		ref		ref		ref	
21–25	14	(6.0)	20	(1.9)		3.360	[1.670; 6.759]	2.954	[1.415; 6.163]	3.301	[1.602; 6.801]	3.031	[1.492; 6.156]	2.822	[1.320; 6.032]
26–30	7	(3.0)	19	(1.8)		1.768	[0.734; 4.260]	1.817	[0.734; 4.502]	1.917	[0.786; 4.675]	1.546	[0.635; 3.765]	1.728	[0.681; 4.383]
> 31	4	(1.7)	8	(0.8)		2.400	[0.716; 8.044]	3.796	[1.045;13.788]	2.807	[0.802; 9.831]	2.136	[0.632; 7.220]	4.562	[1.172; 17.758]

The table shows correlations between infant crying problems and maternal MDI scores, unadjusted, adjusted for socioeconomics, chronic diseases, and previous psychological difficulties. Data were collected three times during pregnancy and once postpartum

Table 3 shows the analysis of the relationship between crying problems in the child and the mother’s anxiety symptoms (expressed as total ASS score) during and after pregnancy and crying problems in the child. Mothers with a high score of anxiety symptoms in first and last trimester of pregnancy and postpartum were more likely to get a baby with crying problems. This tendency remained statistically significant after adjustment for socioeconomic factors, chronic diseases and previous psychological problems of the mothers, but they were less consistent. Similar associations were also observed postpartum, and this tendency remained statistical significant even after adjustments. But they were less consistent even though they remained statistically significant after adjustments.

**Table 3:** Relationship between infant crying problems and maternal anxiety symptoms

	Mothers of infants with crying problems		Mothers of infants without crying problems		Chi-sq test	Unadjusted		Adjusted for socioeconomics		Adjusted for chronic diseases		Adjusted for previous psychological difficulties		Adjusted for all blocks	
	***N*** = 235		***N*** = 1055												
ASS score	N	(%)	N	(%)	***P***-value	OR	[95%CI]	OR	[95%CI]	OR	[95%CI]	OR	[95%CI]	OR	[95%CI]
**First trimester**					0.003										
0–2	116	(49.4)	654	(62.0)		ref		ref		ref		ref		ref	
3–5	66	(28.1)	215	(20.4)		1731	[1.233; 2.430]	1.741	[1.226; 2.472]	1.746	[1.234; 2.469]	1.645	[1.163; 2.326]	1.647	[1.143; 2.372]
6–9	24	(10.2)	98	(9.3)		1.381	[0.848; 2.250]	1.437	[0.854; 2.418]	1.262	[0.754; 2.112]	1.282	[0.779; 2.110]	1.290	[0.748; 2.223]
> 10	29	(12.3)	88	(8.3)		1.858	[1.169; 2.954]	1.924	[1.164; 3.180]	1.971	[1.228; 3.164]	1.642	[1.006; 2.680]	1.742	[1.023; 2.966]
**Second trimester**					0.163										
0–2	139	(59.2)	688	(65.2)		ref		ref		ref		ref		ref	
3–5	44	(18.7)	193	(18.3)		1.128	[0.776; 1.641]	1.163	[0.789; 1.715]	1.126	[0.770; 1.649]	1.049	[0.716; 1.536]	1.060	[0.709; 1.582]
6–9	29	(12.3)	88	(8.3)		1.631	[1.032; 2.578]	1.676	[1.039; 2.704]	1.583	[0.991; 2.531]	1.506	[0.947; 2.397]	1.538	[0.940; 2.515]
> 10	23	(9.8)	86	(8.2)		1.324	[0.807; 2.171]	1.416	[0.842; 2.383]	1.270	[0.756; 2.135]	1.169	[0.702; 1.947]	1.205	[0.694; 2.092]
**Third trimester**					0.007										
0–2	135	(57.5)	683	(64.7)		ref		ref		ref		ref		ref	
3–5	45	(19.2)	221	(21.0)		1.030	[0.712; 1.491]	1.073	[0.731; 1.574]	1.000	[0.684; 1.462]	0.977	[0.671; 1.421]	0.981	[0.661; 1.456]
6–9	38	(16.2)	100	(9.5)		1.923	[1.267; 2.916]	2.088	[1.348; 3.232]	2.065	[1.351; 3.157]	1.774	[1.158; 2.719]	2.007	[1.277; 3.153]
> 10	17	(7.2)	51	(4.8)		1.686	[0.945; 3.009]	1.742	[0.954; 3.183]	1.578	[0.853; 2.919]	1.494	[0.824; 2.712]	1.416	[0.740; 2.710]
**Postpartum**					< 0.001										
0–2	140	(59.6)	803	(76.1)		ref		ref		ref		ref		ref	
3–5	53	(22.6)	160	(15.2)		1.900	[1.327; 2.720]	1.885	[1.296; 2.742]	1.951	[1.350; 2.818]	1.820	[1.260; 2.629]	1.903	[1.287; 2.813]
6–9	22	(9.4)	55	(5.2)		2.294	[1.356; 3.882]	2.315	[1.351; 3.966]	2.374	[1.319; 4.086]	2.180	[1.277; 3.721]	2.195	[1.244; 3.871]
> 10	20	(8.5)	37	(3.5)		3.100	[1.748; 5.498]	3.259	[1.786; 5.946]	3.301	[1.839; 5.925]	2.884	[1.596; 5.211]	3.269	[1.739; 6.147]

This table shows correlations between infant crying problems and maternal ASS scores, unadjusted, adjusted for socioeconomics, chronic diseases, and previous psychological difficulties. Data were collected three times during pregnancy and once postpartum

## Discussion

Women with high scores of both depressive symptoms and anxiety symptoms before birth are in higher risk of having a child with crying problems. The analysis was based on data collected at three different stages of pregnancy and contributes with new knowledge about the association between mental problems at different time points in pregnancy and infant crying. Our study also showed, in line with existing literature, that women with symptoms of depression and anxiety postpartum have a higher risk. The associations appeared to be weakest in the middle of pregnancy. While associations were found for the highest scores, no clear dose-response relationship was found for lower symptom scores.

### Limitations and strengths

Methodological issues relating to the questionnaire instruments and the sampling may affect our results. We measured crying problems using self-reported data from the mother, and the criteria for crying problems were subjective. The amount or intensity of crying may, therefore, vary within the groups, because parents’ perceptions differ. It has been shown that parents’ perception of excessive crying in general is quite reliable when compared to more objective measures, such as audiotaped recordings [42]. The same study found, however, that the reports from some mothers differed from the audiotapes. One might speculate that depressed or anxious mothers are more likely to experience any crying as problematic compared with non-depressed or non-anxious mothers. Our reason for choosing the “problematic” aspect in the crying group was the fact that parents, who find crying problematic, more frequently seek help from the healthcare system, no matter whether the actual time of crying exceeds the “normal” or not.

Self-reported scales were also used to assess depressive symptoms and anxiety symptoms during pregnancy and postpartum [43] (MDI and ASS, respectively). The Edinburgh Postnatal Depression Scale (EPDS) [44, 45] is commonly used to measure postpartum depression, but we chose to use the MDI, as this instrument is widely recognized in general practice in Denmark. ASS is the most frequently used anxiety scale in Danish general practice. We could have used other instruments such as the Beck Anxiety Index (BAI), State-Trait Anxiety Inventory (STAI) or Hospital Anxiety and Depression Scale (HADS) [46] to measure anxiety symptoms, but the ASS was chosen, because this tool is recommended by the Danish College of General Practitioners [47]. The MDI and ASS showed good internal consistency in our study, with Cronbach’s alpha values of 0.84 and 0.80, respectively. None of the scales can be used for diagnostic use and their psychometric properties do not allow for advanced sub-analysis. We, therefore, only used them to classify the women into robust groups.

In this study, there was almost complete data from those who had agreed to participate, and there were few exclusion criteria for participating. However, some selection bias may affect the representativeness of participants. Women not speaking Danish are likely to be underrepresented as all questionnaires were in Danish. We sampled GPs from two representative regions of Denmark, including urban and rural areas, as well as areas with low and high social status and income. This strengthens the geographical and socioeconomic representation, but the GPs could choose not to participate, and the participating GPs might not have asked all eligible women [48]. While this selection bias may have affected estimates of prevalence, it is less likely to have affected the associations investigated.

For the analysis, infant crying was dichotomized into “crying problems” or “no crying problems” though the women’s answers where given on a 3 point scale (major problem, minor problem, no problem). We chose this dichotomization because the distinction between major and minor problems was undefined. Joining the two problematic groups also made sense for statistical reasons because the group with major problems was too small for robust statistical analysis.

We did not control for maternal postpartum mental health when analyzing the data. We were interested in investigating whether mental problems in pregnancy could be an early predictor of crying problems postpartum. A previous study has shown, that women in our study group had depressive symptoms on and off during pregnancy and postpartum [49]. Controlling for symptoms postpartum would therefore lead to overcompensation with respect to our research question in the present study.

### Interpretation of results in relation to other studies

The prevalence of infant crying problems in our study was 18.1%, which is comparable to findings in the literature, where crying problems are described as involving 5–25% of infants depending on the definitions [16, 50, 51].

In accordance with our findings a systematic review from 2018 found strong and consistent evidence for a relationship between maternal depression/anxiety postpartum and excessive crying [24]. Infant crying problems may cause or worsen postpartum depression [52–54], creating a vicious circle, where depressive symptoms and infant crying may reinforce each other. An increasing number of studies have demonstrated that maternal mental health may influence the child’s neuro-behavioral functioning and development [55–57], including emotional regulations, temperament, and crying [58–61], but studies on this subject are still limited, and investigated variables and time points through pregnancy and postpartum differ.

The above review [24] also found that any association between infant crying problems and the mother’s symptoms before birth was less well-established. Our study tries to fill this gap. A few other studies have investigated maternal depression and anxiety in pregnancy and infant crying problems. They have likewise found associations between depressive symptoms during pregnancy and excessive crying [29, 30, 32, 35], and associations between symptoms of anxiety during pregnancy and excessive crying [17, 30, 33]. The studies were performed as prospective cohort studies, but only one of them repeated the questions throughout the pregnancy and postpartum, whereas most of them collected maternal information at only one point during pregnancy. Except for one study [35], none of the studies divided the women into different groups depending on severity of depressive symptoms, but chose one cut-off value when separating the group of women with depressive symptoms from women without.

One study from 1993 did not find any association between prepartum maternal mood and infant crying at 6 weeks [31]. That study’s method differed from ours by using a general questionnaire (the General Health Questionnaire (GHQ)) which was designed to measure nonpsychotic psychiatric disorders, and by dividing mothers into groups of different patterns of distress and not by severity/amount of depressive or anxiety symptoms.

Mechanisms that link mental symptoms during pregnancy to infant crying problems can only be speculative, but recent studies have found a link between prenatal maternal depression and infant outcomes via maternal inflammatory cytokine levels and cortisol, suggesting that biological factors already in pregnancy may influence infant outcomes after birth [59, 60, 62].

Our analyses do not conclude that depressive symptoms and anxiety symptoms during pregnancy are the sole driver of having a child with crying problems; most probably there are other factors that may be more important. Depressive symptoms and anxiety symptoms during pregnancy may not be the direct cause of the crying problems, but merely an (early) sign of some other underlying common cause of both the peripartum symptoms and the crying. The symptoms may be used as indicators of an increased risk of having a child with crying problems and may be used as such to target care.

### Implications for clinical work

Our findings suggest that both depressive symptoms and anxiety symptoms during pregnancy, especially in first and third trimesters, are associated with infant crying problems after birth. While associations were strongest for the highest scores, no clear dose-response relationship was found.

Previous studies have described different challenges associated with infant crying problems, including shaken baby syndrome, child abuse [6–8], and psychosocial problems later in childhood [9–12]. Considering this, and the findings from our study, it is worth asking a few structured questions about the woman’s mental health in the prenatal care consultations. By this, the healthcare system might be able to modify the consequences of infant crying problems after birth.

## Conclusion

We found that women with depressive symptoms and anxiety symptoms before birth are at higher risk of having a child with crying problems. Depressive symptoms and anxiety symptoms during pregnancy may not be the direct cause of the crying problems, but merely an early sign of some other underlying common cause of both the peripartum symptoms and the crying. The symptoms indicate an increased risk of having a child with crying problems. Asking the pregnant women about mental health may promote early intervention with minimal effort.

## Data Availability

The datasets used and analysed during this current study are available from the corresponding author on reasonable request.

## Abbreviations

ASS: Anxiety symptom scale
BAI: Beck anxiety index
EPDS: Edinburgh postnatal depression scale
GHQ: General health questionnaire
GP: General practitioner
HADS: Hospital anxiety and depression scale
MDI: Major depression inventory
STAI: State-trait anxiety inventory
